# Impact of the Mobile HealthPROMISE Platform on the Quality of Care and Quality of Life in Patients With Inflammatory Bowel Disease: Study Protocol of a Pragmatic Randomized Controlled Trial

**DOI:** 10.2196/resprot.4042

**Published:** 2015-02-18

**Authors:** Ashish Atreja, Sameer Khan, Jason D Rogers, Emamuzo Otobo, Nishant P Patel, Thomas Ullman, Jean Fred Colombel, Shirley Moore, Bruce E Sands, HealthPROMISE Consortium Group

**Affiliations:** ^1^Sinai AppLab, Division of GastroenterologyDepartment of MedicineIcahn School of Medicine at Mount SinaiNew York, NYUnited States; ^2^Division of GastroenterologyDepartment of MedicineIcahn School of Medicine at Mount SinaiNew York, NYUnited States; ^3^Frances Payne Bolton School of NursingCase Western Reserve UniversityCleveland, OHUnited States

**Keywords:** medical informatics, patient reported outcome, mHealth, engagement

## Abstract

**Background:**

Inflammatory bowel disease (IBD) is a chronic condition of the bowel that affects over 1 million people in the United States. The recurring nature of disease makes IBD patients ideal candidates for patient-engaged care that is centered on enhanced self-management and improved doctor-patient communication. In IBD, optimal approaches to management vary for patients with different phenotypes and extent of disease and past surgical history. Hence, a single quality metric cannot define a heterogeneous disease such as IBD, unlike hypertension and diabetes. A more comprehensive assessment may be provided by complementing traditional quality metrics with measures of the patient’s quality of life (QOL) through an application like HealthPROMISE.

**Objective:**

The objective of this pragmatic randomized controlled trial is to determine the impact of the HealthPROMISE app in improving outcomes (quality of care [QOC], QOL, patient adherence, disease control, and resource utilization) as compared to a patient education app. Our hypothesis is that a patient-centric self-monitoring and collaborative decision support platform will lead to sustainable improvement in overall QOL for IBD patients.

**Methods:**

Participants will be recruited during face-to-face visits and randomized to either an interventional (ie, HealthPROMISE) or control (ie, education app). Patients in the HealthPROMISE arm will be able to update their information and receive disease summary, quality metrics, and a graph showing the trend of QOL (SIBDQ) scores and resource utilization over time. Providers will use the data for collaborative decision making and quality improvement interventions at the point of care. Patients in the control arm will enter data at baseline, during office visits, and at the end of the study but will not receive any decision support (trend of QOL, alert, or dashboard views).

**Results:**

Enrollment in the trial will be starting in first quarter of 2015. It is intended that up to 300 patients with IBD will be recruited into the study (with 1:1 allocation ratio). The primary endpoint is number of quality indicators met in HealthPROMISE versus control arm. Secondary endpoints include decrease in number of emergency visits due to IBD, decrease in number of hospitalization due to IBD, change in generic QOL score from baseline, proportion of patients in each group who meet all eligible outpatient quality metrics, and proportion of patients in disease control in each group. In addition, we plan to conduct protocol analysis of intervention patients with adequate HealthPROMISE utilization (more than 6 log-ins with data entry from week 0 through week 52) achieving above mentioned primary and secondary endpoints.

**Conclusions:**

HealthPROMISE is a unique cloud-based patient-reported outcome (PRO) and decision support tool that empowers both patients and providers. Patients track their QOL and symptoms, and providers can use the visual data in real time (integrated with electronic health records [EHRs]) to provide better care to their entire patient population. Using pragmatic trial design, we hope to show that IBD patients who participate in their own care and share in decision making have appreciably improved outcomes when compared to patients who do not.

**Trial Registration:**

ClinicalTrials.gov NCT02322307; https://clinicaltrials.gov/ct2/show/NCT02322307 (Archived by WebCite at http://www.webcitation.org/6W8PoYThr).

## Introduction

### Background

Inflammatory bowel disease (IBD) is a chronic condition of the bowel that affects over 1 million people in the United States [1]. Although the incidence of IBD is rising, the precise cause of the disease remains unknown. Medical treatment for IBD has improved significantly in recent years; however, current efforts are largely ameliorative rather than curative. As a result, IBD patients have to cope with a lifelong condition in which there are commonly remissions and relapses. This makes IBD patients the ideal candidates to target for improved self-management when it comes to care.

While diseases such as hypertension and diabetes render themselves well to quality improvement efforts because of standardized indicators such as blood pressure and hemoglobin A1C respectively, a single quality-of-care (QOC) metric cannot define a heterogeneous disease such as IBD, where optimal approaches to manage patients differ between different phenotypes. Furthermore, IBD profoundly affects patients not only physically but also in social, professional, and emotional activities [1,2]. Overall well-being of IBD patients cannot be achieved if these dimensions are not improved [3-6]. Unfortunately, most of the currently proposed quality improvement initiatives in IBD are process measures and do not include quality of life (QOL) or clinically meaningful outcomes such as clinical remission or hospitalizations that matter most to patients and their state of health [7].

Chronic diseases affect almost 1 out of every 2 Americans and produce a significant burden on US health care [8,9]. Meaningful health system quality improvement warrants patient-provider interaction focused on QOC and QOL in chronic diseases like IBD [10,11]. For health care teams, the question remains: how do we better engage patients without placing increased time constraints on health care staff? Based on pilot work, we believe that patients are as eager as physicians, if not more, to improve their QOL and care, and involving them as partners to improve care can bring remarkable efficiency to current quality improvement efforts [3].

### Objectives

**Figure 1:**
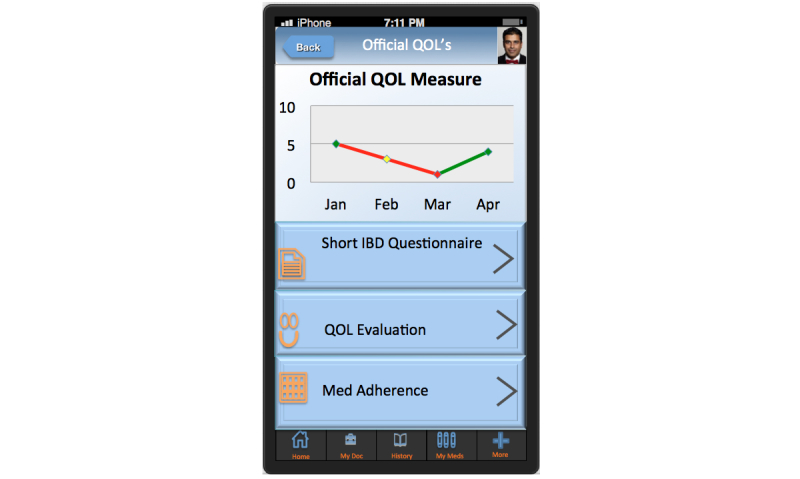
Quality of life measure.

**Figure 2:**
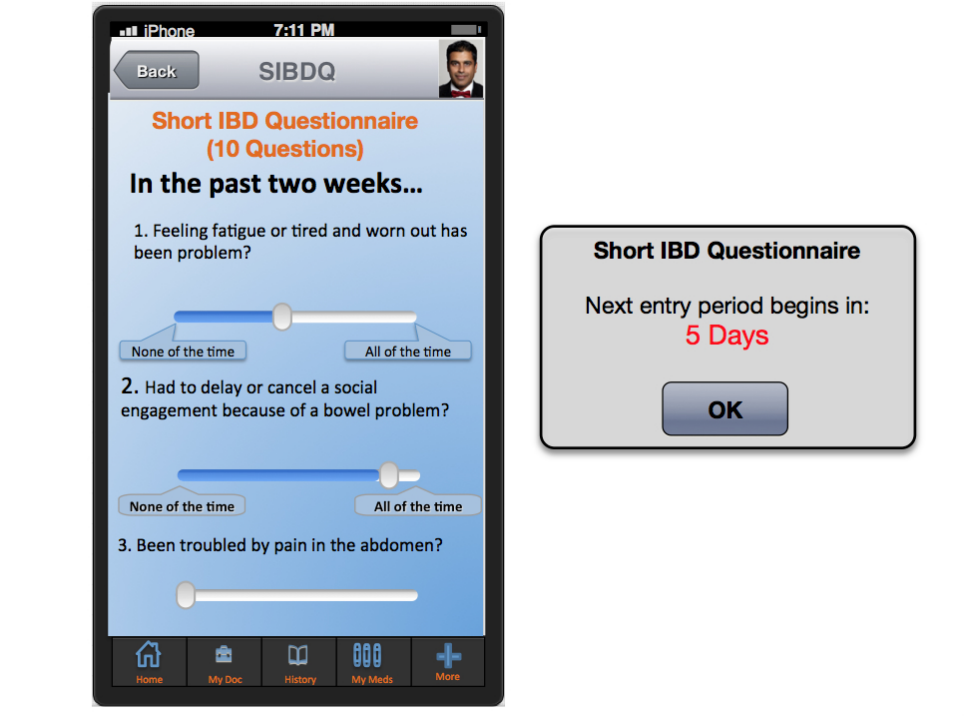
Short Inflammatory Bowel Disease Questionnaire.

HealthPROMISE [12] is a unique cloud-based PRO (patient-reported outcome) and decision support platform developed at Sinai AppLab, Icahn School of Medicine at Mount Sinai [13] Patients track their QOL and symptoms, and providers can use the visual data in real time (integrated with electronic health records [EHRs]) to provide better care to their entire patient population (Figures 1 and 2). HealthPROMISE addresses unique challenges to improving quality and outcomes for patients with a chronic disease like IBD.

### Adopting a Broader Definition of Quality

**Figure 3:**
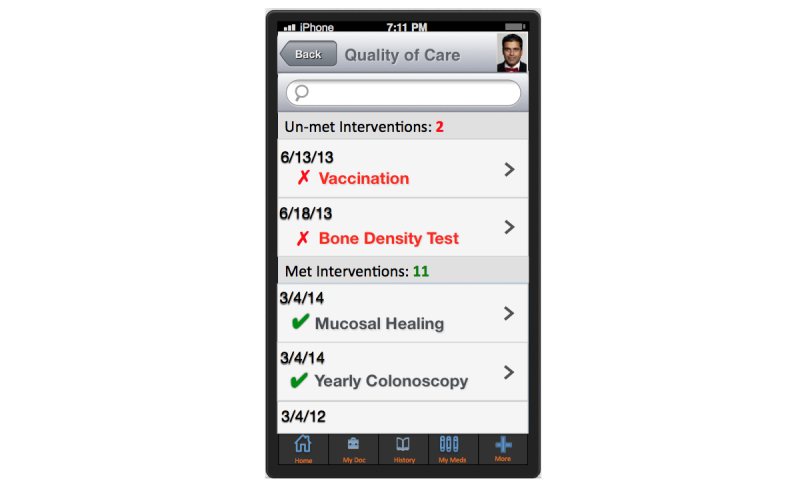
Quality of care.

A more comprehensive assessment may be provided by complementing the QOL with quality of care metrics (Figure 3). QOL has been defined as “a global measure of patient’s perceptions, illness experience, and functional status that incorporates social, cultural, psychological, and disease-related factors” [14]. QOL metrics can be used to inform outcomes in clinical encounters, monitor population health, and as end points in clinical trials [14]. National Institutes of Health (NIH) Patient Reported Outcomes Measurement Information System [15] and more recently Project Health Design [16] have provided valuable insights into generic measurements for QOL. To address this challenge, we have previously defined a set of comprehensive quality indicators for IBD patients through analyzing different focus groups to study what factors patients assess and value when defining “quality” in terms of living with IBD and the treatment of IBD. Additionally, through semi-structured interviews and Delphi panel sessions with 15 providers, provider input on QOC was recorded.

### Decreasing the Burden of Measuring Quality

Quality improvement efforts so far have shown that measuring even limited QOC metrics carries a prohibitively high administrative and cost burden. The estimated costs from the Institute for Healthcare Improvement quality improvement initiative for either congestive heart failure or diabetes ranged from $81,000 to $148,000 per organization [17]. Chen and Bates have shown that total reported costs for inpatient quality improvement for a hospital ranged from $2 million to $21 million, with the majority of costs attributed to collecting and reporting quality metrics for national organizations [18]. This burden of measuring quality is likely to increase exponentially when multiple QOC metrics are included in quality measurement. To address this challenge, patient- and physician-provided indicators were incorporated into a mobile health strategy platform, HealthPROMISE, that allows patients to record and self-report their QOL and treatment with regards to their IBD.

### Improving the Effectiveness of Quality Improvement Initiatives

Currently, there is no well-accepted national model for quality improvement. Most of the quality improvement projects to date involve some kind of data abstraction from the clinical encounters that is fed into a registry to allow benchmarking, risk adjustment, and quality reporting. This cycle takes anywhere from a few weeks to a few months and happens long after the patient has left the health care facility. Patients are not involved in measuring or improving quality. Thus, an important patient-physician “productive interaction” opportunity to improve outcomes at the point of care is missed [19]. In HealthPROMISE, patients track symptoms and QOL before office visits and in waiting rooms, thus allowing meaningful discussion about QOC to take place during office visits (Figures 1 and 2).

The aim of this research protocol is to evaluate the patient-centric Web- and mobile-based application, HealthPROMISE, where IBD patients longitudinally measure their QOC and QOL metrics and physicians use this information for collaborative decision making and improving patient outcomes. Our hypothesis is that a patient-centric self-monitoring and collaborative decision support platform will lead to sustainable improvement in overall QOL for IBD patients.

## Methods

### Study Design

**Figure 4:**
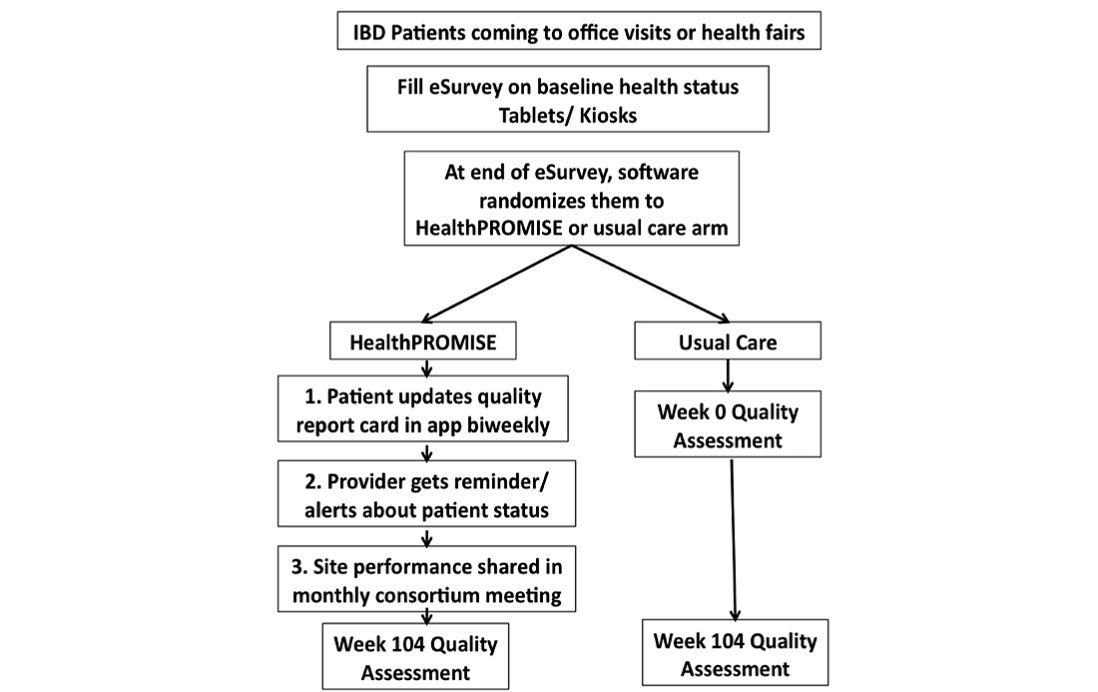
Point of care recruitment and randomization.

**Figure 5:**
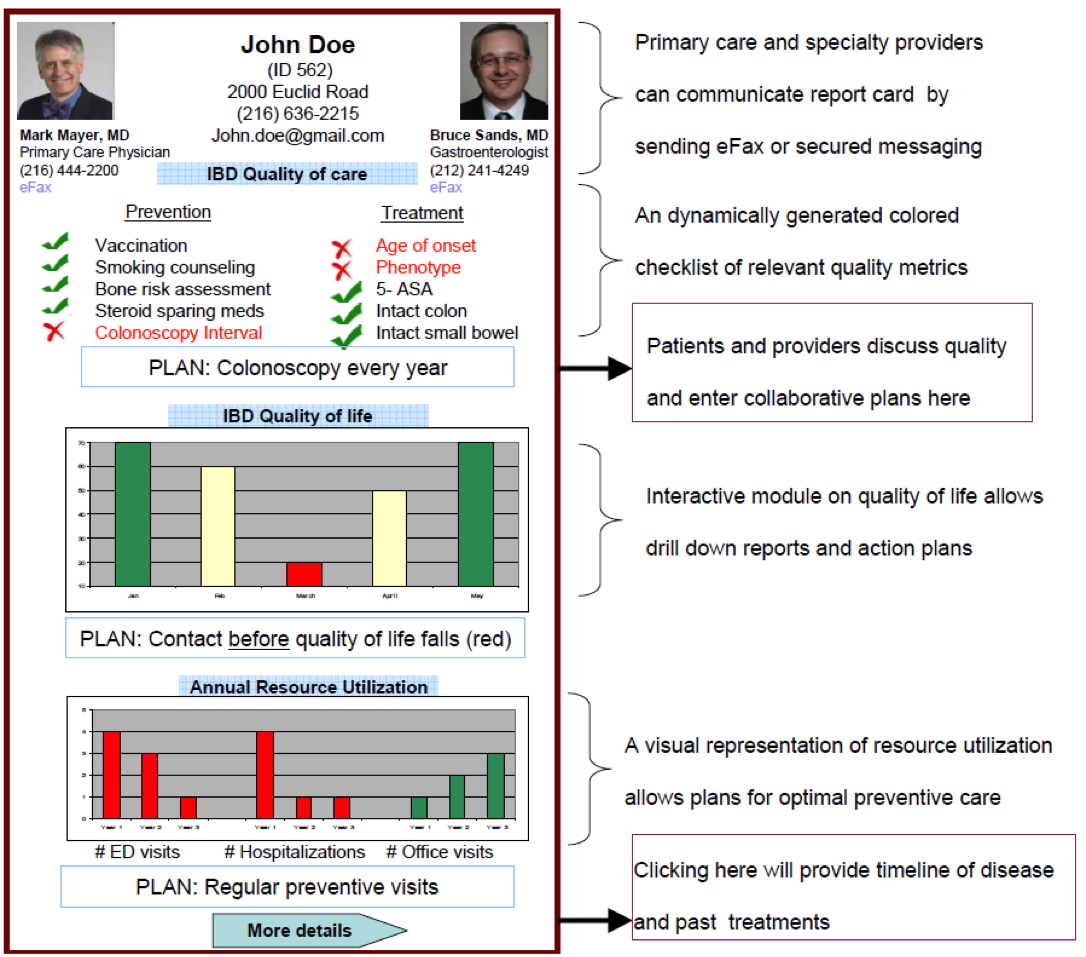
Shows initial mock-up of app.

This is a phase III, single-center, pragmatic randomized controlled trial (RCT) to evaluate if a patient-centric self-monitoring and collaborative decision support platform will lead to sustainable improvement in overall QOL for IBD patients. It is intended that 300 patients with IBD will be recruited into the study (allocation ratio 1:1; Figure 4). After meeting all the inclusion criteria with no exclusions, patient is asked to complete a tablet-based screening questionnaire at the end of which patient is randomized at the point of care to intervention or control arm. Patients in the control arm will receive an IBD education app PIN whereas the intervention arm will receive HealthPROMISE app PIN (Figure 3). Intervention patients enter their data once every 2 weeks and this data is then made visible to providers using a Web-based dashboard integrated with the EHR (Figures 4 and 5). Intervention and control apps will be provided free of charge to patients, and patients will be given $25 after completing initial and end of study questionnaires.

### Study Population

Patients will be recruited at outpatient and inpatient facilities in an academic center through informational paper and electronic flyers. Once enrolled, patients will receive a walkthrough of the app, which includes access to a training video. The provider dashboard will also have access to the training video. Eligible patients will be 18 years or older, have a mobile phone or access to the Internet at home, and be able to complete a Web-based questionnaire in English. Exclusion criteria include the inability to communicate with the investigators and comply with the study requirements, presence of short bowel syndrome or stoma, and presence of a condition or disease that, in the opinion of the investigators, may make it difficult for the patient to use the HealthPROMISE app, including, but not limited to, advanced dementia.

### Study Instruments

#### Overview

**Figure 6:**
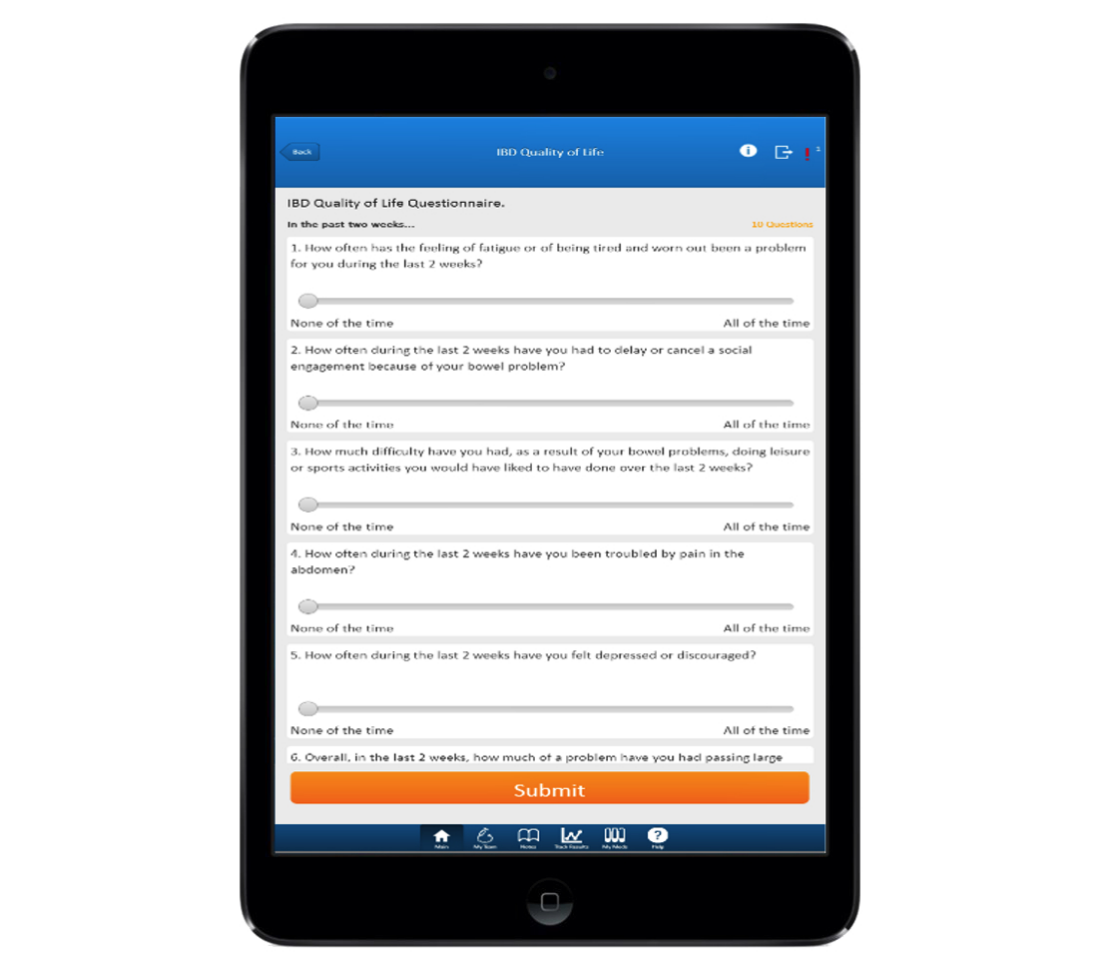
Shows completed app.

A combination of different questionnaires (eg, SIBDQ), symptom updates, and quality indicators relevant for evaluating patient status will be the data collected during this study through the HealthPROMISE app (Figure 6).

#### Disease Specific Quality of Life Questionnaire

The Short Inflammatory Bowel Disease Questionnaire (SIBDQ) [20] is a validated and reliable tool to measure health-related QOL in adult patients with IBD. The questionnaire consists of 10 questions scored in four domains: bowel symptoms, emotional health, systemic systems, and social function. The SIBDQ is a respected QOL questionnaire used extensively in academic research and clinical trials. Study patients in the control arm and interventional arm will complete an SIBDQ as part of a survey to objectively measure QOL at baseline and at exit (52 weeks or 104 weeks). Additionally, patients in the intervention arm will be asked to complete the SIBDQ every 2 weeks; this will be used to classify patients as having “good control,” “fair control,” or “poor control.”

#### General Quality of Life Questionnaire

EQ-5D is a standardized instrument for measuring generic QOL [21]. Applicable to a wide range of health conditions and treatments, it provides a simple descriptive profile and a single index value for health status. EQ-5D is primarily designed for self-completion by respondents. It is cognitively simple and takes only a few minutes to complete. It is generally recognized that a change of 0.5 points (on a scale of 1-7) is the minimal clinically important difference (MCID), consistent with moderate effect size. Patients in the intervention arm will be asked to complete the EQ-5D every 2 weeks.

#### Other Instruments

eHEALS is an 8-item measure of eHealth literacy developed to measure consumers’ combined knowledge, comfort, and perceived skills at finding, evaluating, and applying electronic health information to health problems [22]. This instrument has been psychometrically validated and its score positively correlated with intention to use personal health records. Patient Activation Measure (PAM-13) will be used to measure patient activation and engagement with health [23]. eHEALS and PAM-13 will be completed by patients in both arms during entry and exit surveys only.

**Figure 7:**
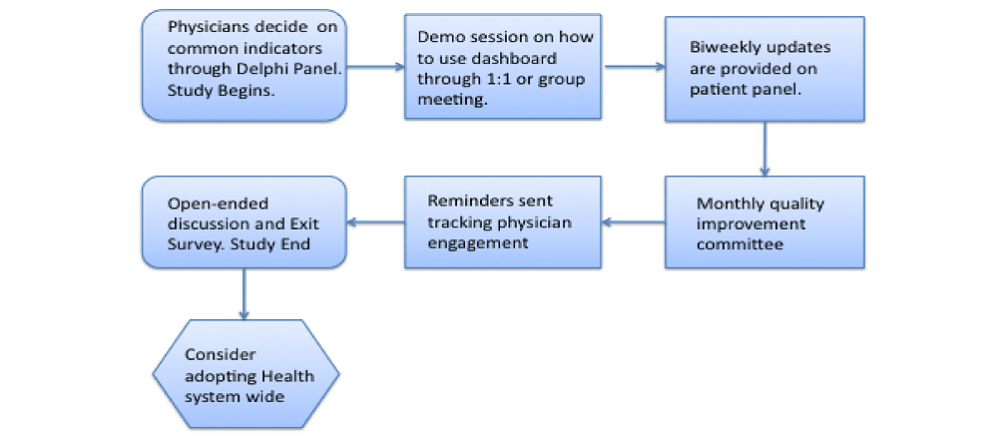
Provider workflow on app dashboard.

Quality indicators are included from a list of indicators published by national societies and finalized through a Delphi panel of IBD providers [24,25]. These will be updated every three months by either providers or patients, along with hospitalization and emergency department visit information (Figure 7).

Utilization will be assessed through log-in, page views, health information updates, and response to alerts and reminders.

## Results

### Outcome Measures

All outcome data (Textbox 1) will be collected online. Additionally, we will conduct subgroup analysis of patients with poor disease control at week 0 (SIBDQ≤30) and in those with high patient-reported anxiety, depression, or stress achieving primary and secondary endpoints. We will assess change in inflammatory markers, endoscopic scores, and additional quality metrics in each group and determine predictors of HealthPROMISE and control app utilization and its impact on other primary and secondary end-points. Consistent with the pragmatic nature of the trial, study progress will be assessed throughout and effort will be optimized to better engage providers and patients (Table 1).

**Primary and secondary endpoints.**

Primary endpoint

- Number of quality indicators met in HealthPROMISE versus control arm

Secondary endpoints

- _Decrease in number of emergency visits due to IBD_
- _Decrease in number of hospitalization due to IBD_
- _Change in generic QOL score (EQ-5D) from baseline_
- _Proportion of patients in each group who meet all eligible outpatient quality metrics_
- _Proportion of patients in disease control in each group_
- _Emergency visits in each group_
- _Hospitalizations in each group_
- _General QOL scores in each group_
- _Per protocol analysis of intervention patients with adequate HealthPROMISE use (more than 6 log-ins with data entry from week 0 through week 52) achieving above mentioned primary and secondary endpoints_

**Table 1:** Evaluation metrics for HealthPROMISE progress

Metric		Target Group	Goal / Timeline
**Process**			
	Number of providers trained	Investigators	10 in 2 months
	Number of patients enrolled	Participants	300 in 6 months
	Recruitment and training of key personnel	Coordinator	1 in 3 months
	Patient utilization of HealthPROMISE	Participants	Ongoing
	Provider utilization of HealthPROMISE	Providers	Ongoing
	Response to alert within 2 business days	Providers	>90%
**Outcome**			
	Improvement in quality of care metrics	Provider, Center	Quarterly reports
	Improvement in quality of life	Provider, Center	Quarterly reports
	Readmission rate in two arms	Provider, Center	Quarterly reports

### Statistical Analysis Plan

#### Overview

We will use SAS 9.2 (SAS Institute, Inc) to calculate frequencies and percentages for categorical factors and means with standard deviations and/or percentiles for continuous factors. Pearson’s chi-square tests will be used for primary outcome (number of quality indicators met in HealthPROMISE vs control arm) and secondary outcomes. We will calculate percentage score for each patient at baseline and at week 104 ([number of quality metrics met/quality metrics eligible]*100). Change in the percentage score from baseline to week 104 will be aggregated for each arm to calculate percentage-point improvement in quality metric, similar to the strategy by Cebul et al [26].

Analysis of covariance (ANCOVA) will be performed to assess differences in the area under the curve of QOL scores while adjusting for baseline QOL score. To assess the association between patient and practice characteristics and achievement of eligible quality metrics, we will use multivariable analyses. Since the data will be hierarchically structured, with patients clustered within physicians and metrics clustered within patients, we will construct multilevel, generalized, linear mixed models with random effects to determine predictors of quality care, similar to the strategy used by Kanwal et al [27]. Independent variables will include demographic characteristics (age, gender), education and income level, race and ethnicity, computer usage, eHealth literacy scores, clinical characteristics (comorbidities, phenotype, disease severity), and provider characteristics (gender, age, site of practice, years of practice, presence of nurse practitioner).

#### Interim Analysis

Interim analysis will be performed once 150 patients are followed up for week 52. If primary outcome is met by that time, all patients will be offered HealthPROMISE app and followed for the additional 52 weeks.

#### Sample Size Justification

Study endpoints will be primarily assessed using intention-to-treat (ITT) analysis; however, per-protocol analysis will also be performed. The study is to be powered such that there is a>80% probability of demonstrating a difference with a *P* value *(P* = .05) using a two-tailed *t* test.

We assume that 128 out of 150 subjects (85%) in the intervention arm will meet all quality indicators (primary outcome) and expect that this percentage will be at least 15% lower in the control arm. A sample size of 95 patients will be needed in each arm to achieve at least 80% power to detect the difference with a 5% one-sided significance level.

Accounting for an estimated 30% attrition rate, we will require a total of 250 IBD patients to be enrolled in the study. Since some patients may agree to enroll but not download the app or use the PIN, we will recruit a total of 300 patients in the study.

For secondary outcomes related to QOL, the control arm is not a placebo arm and physicians are free to initiate any therapy based on patients' symptoms. Hence, we will assess the difference in proportion of patients achieving MCID in the HealthPROMISE arm versus control arm in the study. Using the distribution-based approach, an effect size of 0.5 SD is the closest estimate for determining MCID for SIBDQ and EQ-5D. Assuming that 20% more patients in HealthPROMISE arm will achieve MCID than in the control arm, the sample size of 250 patients will have 88% power to detect the difference with a 5% one-sided significance level and an estimated 30% attrition rate.

## Discussion

### Principal Findings

This pragmatic trial will help us study if a patient-centric self-monitoring and collaborative disease management app and dashboard can lead to improvement in care provided to IBD patients. Our hypothesis is that IBD patients using the HealthPROMISE platform will have significant improvement in QOC metrics, QOL, and resource utilization by the end of the 2-year study period when compared to IBD patients in the control arm (using a health education app alone).

### Future Direction and Sustainability

HealthPROMISE can be a sustainable platform in the long run because it is patient-centric, device and disease agnostic, and not dependent on proprietary EHRs. As most of the data is entered by patients, the cost of running, supporting, and sustaining HealthPROMISE is very low compared to traditional disease registries. HealthPROMISE has a rapid form generator capability to allow it to be customized for other chronic diseases. Additionally, the decision support that generates alerts and dashboard reports is within the stand-alone HealthPROMISE app and not dependent on proprietary EHRs. We aim to integrate HealthPROMISE with personal health records, partner with national societies, and support through consortia so it can become a new standard of quality care for IBD and other chronic diseases.

### Conclusion

HealthPROMISE is a unique cloud-based PRO and decision support tool that empowers both patients and providers. Patients track their QOL and symptoms, and providers can use the visual data in real time (integrated with EHRs) to provide better care to their entire patient population. Using pragmatic trial design, we hope to show that IBD patients who participate in their own care and share in decision making have appreciably improved outcomes when compared to patients who do not [28,29].

## Multimedia Appendix 1

Initial critique of the proposal before it got funded in second attempt.

## Multimedia Appendix 2

Informed consent form.

## Multimedia Appendix 3

CONSORT-EHEALTH checklist V1.6.2 [30].

## Abbreviations

IBD: inflammatory bowel disease
QOL: quality of life
QOC: quality of care
PRO: patient-reported outcome
EHR: electronic health records
NIH: National Institutes of Health
RCT: randomized controlled trial
SIBDQ: Short Inflammatory Bowel Disease Questionnaire
MCID: minimal clinically important difference
PAM: patient activation measure
