# Supplementary material for: Impact of the Mobile HealthPROMISE Platform on the Quality of Care and Quality of Life in Patients With Inflammatory Bowel Disease: Study Protocol of a Pragmatic Randomized Controlled Trial
Source: JMIR Res Protoc. 2015 Feb 18;4(1):e23. doi: 10.2196/resprot.4042 (PMC4376196; doi:10.2196/resprot.4042)
Supplement: Supplementary file 1 [file resprot_v4i1e23_app1.pdf]

SUMMARY STATEMENT
(Privileged Communication)

PROGRAM CONTACT:
Dr. JUDITH PODSKALNY PhD
(301) 594-8876
podskalnyj@extra.niddk.nih.gov

Release Date: **07/28/2012**

Application Number: **1 K23 DK097451-01**

Principal Investigator

ATREJA, ASHISH

Applicant Organization: MOUNT SINAI SCHOOL OF MEDICINE

Review Group: **DDK-C**
Digestive Diseases and Nutrition C Subcommittee

Meeting Date: **06/27/2012**
Council: **OCT 2012**
Requested Start: **09/01/2012**

RFA/PA: **PA11-194**
PCC: **NJP DDTR**

Project Title: **IBDPROMISE: A Web-based Patient-centric Model to Improve Quality and Outcomes**
SRG Action: **Impact/Priority Score: 30**
Human Subjects: **30-Human subjects involved - Certified, no SRG concerns**
Animal Subjects: **10-No live vertebrate animals involved for competing appl.**
Gender: **1A-Both genders, scientifically acceptable**
Minority: **1A-Minorities and non-minorities, scientifically acceptable**
Children: **1A-Both Children and Adults, scientifically acceptable**
Clinical Research - not NIH-defined Phase III Trial

Project Year	Direct Costs Requested	Estimated Total Cost
1	163,150	175,652
2	164,006	176,574
3	163,438	175,962
4	163,438	175,962
5	164,713	177,335
TOTAL	818,745	881,485

ADMINISTRATIVE BUDGET NOTE: The budget shown is the requested budget and has not been adjusted to reflect any recommendations made by reviewers. If an award is planned, the costs will be calculated by Institute grants management staff based on the recommendations outlined below in the COMMITTEE BUDGET RECOMMENDATIONS section.

ADMINISTRATIVE NOTE

1K23DK097451-01 Atreja, Ashish

SCIENTIFIC REVIEW ADMINISTRATOR'S NOTES

RESUME AND SUMMARY OF DISCUSSION: An application for a Mentored Patient-Oriented Research Career Development Award (Parent K23) was submitted by the Mount Sinai School of Medicine on behalf of Dr. Ashish Atreja. The research goal “centers on the development of a web-based application in which patients with inflammatory bowel disease enter information about their quality of life and quality of care with the goal of using such data for quality improvement.” Strengths of the application include a motivated, well-trained and productive candidate; a strong mentoring team with complimentary expertise; an outstanding research and training environment; good institutional support; an innovative research proposal; and a research topic of clinical significance. In addition to these strengths, concerns were also noted, including weaknesses in the research plan; the need for additional mentors with “expertise on assessing clinical operational factors and effectiveness of the QI activity, and for ongoing informatics issues”; a question as to whether the mentor/co-mentor relationships will be intense and productive; and a lack of consideration of the generalizability of this web-based application developed within a highly specialized tertiary care facility to general IBD practice. The overall merit of this application is considered to be excellent, and five years of support are recommended.

DESCRIPTION (provided by applicant): Background: Measuring and improving healthcare quality is an important national challenge. The discussion about quality and disease management rarely happens during office visits when patients and providers have a unique opportunity to listen to each other and make collaborative decisions. Hypothesis: Encouraging patients to proactively measure and improve quality can bring remarkable efficiency, effectiveness and cost-savings to quality improvement efforts. Goal: To acquire skills to design and evaluate a patient-centric model, IBDPROMISE (IBD Patient Reported Outcomes and Medical Illness Severity Evaluation), in which patients with inflammatory bowel disease (IBD) measure their quality of care metrics (such as colonoscopy surveillance) as well as quality of life in waiting rooms and at home; and physicians use this information at the point of care for quality improvement efforts. The specific aims of the project are: " AIM I. Define a set of comprehensive quality of care metrics (such as immunization, appropriate colonoscopy surveillance) for IBD patients from national recommendations. " AIM II. Measure longitudinally quality of care metrics and quality of life (QOL). " AIM III. Conduct a randomized controlled trial to determine the impact of IBDPROMISE in improving outcomes (quality of care, quality of life, patient adherence, disease control and resource utilization). Methods: All eligible adult patients arriving in Mount Sinai Hospital IBD center during study recruitment will be offered tablet-based questionnaire in waiting rooms and enrolled once consented. After a baseline period (at least six months), participants in the IBDPROMISE arm will be asked to report their quality of care metrics and QOL every month for 18 months. This will generate an electronic health card (detailing disease summary, quality metrics and a graph showing the trend of QOL and healthcare utilization over time). Patients and providers will use the health card, active decision support (a drop in scores) and passive decision support (quality dashboards and reminders) to improve the quality of care collaboratively. Outcome: The primary end-point of the study is the proportion of patients in each group (IBDPROMISE vs. control) who meet all eligible quality metrics at week 104. Secondary endpoints will be comparison of quality of life, disease control rates and utilization of healthcare resources between the two arms. Future Direction: This project will help me acquire critical skills in self-management, quality improvement, design of clinical trials and analysis of quality of life data and cost effectiveness. In the future, IBDPROMISE and its point of care QI model within EHR, if proven successful, will be made available license-free to other academic institutions, and help me implement multi-site clinical trials and prospective cohort studies in IBD.

PUBLIC HEALTH RELEVANCE: Measuring and improving healthcare quality is an important national challenge. Improvement cannot be achieved if patients are not made aware of care quality that they are

receiving, hence we are providing electronic Quality Report Cards to patients and providers that will bring discussion about quality and outcomes at the forefront of care and help improve patient outcomes.

CRITIQUE 1:

Overall Impact:

This productive and informatics-expert clinician-investigator is presenting a QI project that is innovative in methodology, high in significance for advancing IBD care, and robust as a training vehicle for the applicant. The shortcomings, which are all addressable, concern rounding out the mentor group, and refining and augmenting the research plan.

1. Candidate:

Strengths

- Strong and productive prior training and expertise in medical informatics, notably procedure-based practice assessment, cost effectiveness, and web-portal based patient interfaces
- Consistently productive publication track record in each of his areas of research, with 31 listed publications, many first authored in specialty clinical and clinical research journals.
- Designed, funded, and performed an ACG pilot project which introduces the research direction of the present application

Weaknesses

- None noted

2. Career Development Plan/Career Goals & Objectives/Plan to Provide Mentoring:

Strengths

- The plan complements with and distinguishes from his prior training.
- It is granular and well-integrated with the research plan (patient self-management, QI, and clinical trials strategies and methodologies).
- Mentors and advisors are relevant to the career development plan

Weaknesses

- There are some gaps in mentoring scope, and the remote-mentoring needs to be validated (see below)

3. Research Plan:

Strengths

- The original design of the project was piloted as CrohnsPROMISE, a funded and implemented project of the applicant, which has established feasibility, and identified areas for refinement.
- This QI project is innovative and important, by elaborating a patient self-reporting element, and alert to validation issues on such reporting (which were probed in the pilot project)..
- It is important in developing intermediate outcomes (e.g., QALY and QOL), as well as process outcomes.
- Feedback elements are important features for a QI initiative, and several (alerts, report card, dashboards) are introduced and tested here

Weaknesses

- Cultural, technologic, and language (level and Spanish/English) acceptability assessment for the self-reporting elements should be developed and introduced into the study plan
- Impact and feasibility of the process on clinical office/physician workflow and resources should be a study element
- The differences in how this would work in a referral center versus community practice setting should be more deeply considered, either by fully integrating a community site, or by delineating what the limitations might be in analysis restricted to MSSM.

4. Mentor(s), Co-Mentor(s), Consultant(s), Collaborator(s):

Strengths

- Drs. Sands and Moore are outstanding experts and sophisticated and productive mentors for clinical trials and patient self-assessment, respectively.
- A network of advisors is an additional resource for mentorship and consultation, such as Drs. Ullman at MSSM, and Drs. Siegel and Kappleman (Dartmouth and UNC).

Weaknesses

- It is likely but not yet established that the mentor/co-mentor relationships will be intense and productive (Dr. Moore in Cleveland, and Dr. Sands not previously a mentor for the applicant, and highly committed as division chief and with intense and diverse research commitments).
- Mentors with expertise on assessing clinical operational factors and effectiveness of the QI activity, and for ongoing informatics issues (more than claiming a connection to the CIO or operations team)

5. Environment and Institutional Commitment to the Candidate:

Strengths

- MSSM has an outstanding IBD clinical center, clinical research infrastructure, and outstanding clinical and clinical research expertise to draw upon
- The applicant has a new full faculty appointment, and relevant resource, protected time, and personal commitment at the division and department levels.

Weaknesses

- None noted

Protections for Human Subjects:

Acceptable Risks and Adequate Protections

Data and Safety Monitoring Plan (Applicable for Clinical Trials Only):

Not Applicable (No Clinical Trials)

Inclusion of Women, Minorities and Children:

G1A - Both Genders, Acceptable

M1A - Minority and Non-minority, Acceptable

C3A - No Children Included, Acceptable

Vertebrate Animals:

Not Applicable (No Vertebrate Animals)

Biohazards:

Not Applicable (No Biohazards)

Training in the Responsible Conduct of Research:

Acceptable

Comments on Format (Required):

- on-line and FTF coursework

Comments on Subject Matter (Required):

- CITI, ethical, policy, and stem cell

Comments on Faculty Participation (Required; not applicable for mid- and senior-career awards):

- faculty direct coursework; Dr. Sands (mentor) oversees this training

Comments on Duration (Required):

- quarter year, and annual

Comments on Frequency (Required):

- biweekly, and 1-2 sessions

Select Agents:

Not Applicable (No Select Agents)

Resource Sharing Plans:

Acceptable

- There should be a further explanation on how the tools will be made available

Budget and Period of Support:

Recommend as Requested

CRITIQUE 2:

Candidate: 2
Career Development Plan/Career Goals /Plan to Provide Mentoring: 2
Research Plan: 3
Mentor(s), Co-Mentor(s), Consultant(s), Collaborator(s): 2
Environment & Commitment to the Candidate: 2

Overall Impact:

Dr. Ashish Atreja has a unique background in informatics which establishes the foundation for pursuing the use of QI tools in IBD management. This proposal will pilot a web-based application for IBD patients in an effort to ultimately improve QOL and care, which has the potential to provide a useful supplement to patient care. Dr. Atreja's new faculty position at Mt. Sinai will provide an outstanding venue for piloting this tool, and he has assembled an outstanding team of mentors. Additional information on expectation in quantitative outcomes, as well as areas of potential bias and details on design would enhance the proposal.

1. Candidate:

Strengths

- Dr. Atreja's training in informatics provides an excellent opportunity to apply important methods and tools to patient care. Dr. Atreja is a 1st author on a number of studies addressing IBD guideline compliance, cost-effectiveness of selected interventions, and utilization of web-based training (Am J Infect Control, BMC Med Educ, ScientificWorldJournal, Dig Dis Sci).
- Grant from the ACG provided the basis for testing the feasibility and utilization of CrohnsPROMISE.

Weaknesses

- None

2. Career Development Plan/Career Goals & Objectives/Plan to Provide Mentoring:

Strengths

- A career plan is well-delineated. Dr. Atreja will attend courses to assist in the skills needed to pursue the project proposed, including didactics in self-management, QI, and clinical trial methodology.

Weaknesses

- None

3. Research Plan:

Strengths

- Establishing and validating a web-based application that involves IBD patients in improving their QOL and care provides for a potentially important, systematic mechanism to improve IBD management outcomes.
- Preliminary data in 126 CD patients demonstrates the ability of patients to enter their data, with good concordance in most measures except phenotype and bone scan results.
- Aim I will define the quality indicators for IBD patients to be ultimately included in the assessment. Systematically reviewing the indicators recommended by national societies combined with potentially additional indicators is a reasonable approach.
- Aim II to improve the design of IBDPROMISE is a critical component of optimizing this tool. The key improvements described are very appropriate and important aspects of the development.
- Aim III will conduct a randomized controlled trial to determine the impact of IBDPROMISE in improving outcomes. This is ultimately essential to determining efficacy. The number of patients needed for enrollment is feasible given the estimates of the patient volume in the IBD center.

Weaknesses

- Additional information on target numbers for participation in the focus groups (both patients and physicians), and for experts to participate in questionnaires for final consensus should be included and justified.
- How will efficacy of the improvements implemented in Aim II be assessed?
- Addressing potential areas of bias would be beneficial. For example, physicians will not be blinded to the study arm (they will receive alerts or dashboard access for those patients enrolled in the IBDPROMISE arm).
- Aim III sample size calculation, it's unclear what the expected difference in outcome is between the intervention and control arm. An estimate in the difference in each of the goals listed (improved quality of care metrics, improved quality of life and reduction in costs) would be helpful, as would examples of overall improvement in other diseases with such interventions.
- Additional details aspects of the design would be beneficial. For example, how often are patients asked to make entries online or is this adlib, and how will differential frequencies of interactions be considered in interpretation?

4. Mentor(s), Co-Mentor(s), Consultant(s), Collaborator(s):

Strengths

- Dr. Bruce Sands at Mount Sinai in NY will serve as Dr. Atreja's mentor; Dr. Atreja will be joining the faculty at Mt. Sinai in July, 2012. Dr. Sands is a widely-recognized expert in the field of IBD and Chief of GI at Mount Sinai. Dr. Sands has been interacting with Dr. Atreja on the current proposal despite the geographical distance.
- Professor Shirley Moore at Case Western Reserve, with whom Dr. Atreja has an ongoing relationship, will serve as Dr. Atreja's co-mentor. Dr. Moore has a PhD in nursing, is Associate Dean for Research and is a leader in quality and safety in healthcare, including in patient self-management.

Weaknesses

- None

5. Environment and Institutional Commitment to the Candidate:

Strengths

- Mount Sinai has an outstanding IBD Center that will provide the basis for patient recruitment. Dr. Sands indicates a commitment of the IBD faculty to enroll patients, interaction with Dr. Thomas Ullman, who has a focus on QI in IBD, and the support of key informatics personnel at Mount Sinai.

Weaknesses

- None

Protections for Human Subjects:

Acceptable Risks and Adequate Protections

Data and Safety Monitoring Plan (Applicable for Clinical Trials Only):

Acceptable

Inclusion of Women, Minorities and Children:

G1A - Both Genders, Acceptable

M1A - Minority and Non-minority, Acceptable

C3A - No Children Included, Acceptable

Vertebrate Animals:

Not Applicable (No Vertebrate Animals)

Biohazards:

Not Applicable (No Biohazards)

Training in the Responsible Conduct of Research:

Acceptable

Comments on Format (Required):

- CITI certificate and completion of 4hr seminar on ethical conduct as part of MPH.

Comments on Subject Matter (Required):

- Plan for CITI re-certification; 'Responsible Conduct of Research' course at Mt. Sinai and as required by IRB.

Comments on Faculty Participation (Required; not applicable for mid- and senior-career awards):

- Dr. Bruce Sands will monitor.

Comments on Duration (Required):

- CITI valid for 2 yrs; 8 sessions in 'Responsible Conduct of Research' course in year 1

Comments on Frequency (Required):

Select Agents:

Not Applicable (No Select Agents)

Resource Sharing Plans:

Unacceptable

- A formal attachment indicating plans for resource sharing should be included (mention is made in the main proposal).

Budget and Period of Support:

Recommended budget modifications or possible overlap identified:

- Additional clarity for the 'Other costs' in year 2 listed to be provided.

CRITIQUE 3:

Candidate: 2

Career Development Plan/Career Goals /Plan to Provide Mentoring: 2
Research Plan: 3
Mentor(s), Co-Mentor(s), Consultant(s), Collaborator(s): 3
Environment & Commitment to the Candidate: 2

Overall Impact:

This is an innovative and interesting proposal by a motivated and talented gastroenterology junior faculty member who is cross-trained in medical informatics. The proposal centers on the development of a web-based application in which patients with IBD enter information about their quality of life and quality of care with the goal of using such data for quality improvement. The strengths of the proposal include a novel area of research and development of a potentially useful tool for IBD patient management and assessment of quality outcomes, an increasing imperative. As a training vehicle, the proposal also nicely blends several different research efforts, including qualitative research (e.g. focus groups), measurement of quality metrics using data obtained from these focus groups, and conduct of a clinical trial to examine the impact of this application on several IBD outcomes. The applicant is outstanding, with a strong track record in informatics. The mentors are also outstanding, combining clinical research expertise in IBD with patient-centered management, although it is notable that there is not a medical informatics mentor included. Weaknesses of the application include a lack of consideration of the generalizability of this web-based application developed within a highly specialized tertiary care facility to general IBD practice; utility of this application for patients of varying disease severity; inability to include populations with lack of access to technology or without technology literacy; and clear discussion of how this will be integrated into electronic health records. The applicant is changing institutions at a critical juncture in his research career development and aligning himself with a new mentor, which does raise concerns.

1. Candidate:

Strengths

- Motivated, well-trained junior faculty member with background in medical informatics
- Strong publication record in informatics related research
- Enthusiastic mentors
- Successful recipient of an ACG pilot award

Weaknesses

- None noted

2. Career Development Plan/Career Goals & Objectives/Plan to Provide Mentoring:

Strengths

- Well-outlined career development plan which integrates formal coursework into a cohesive curriculum.

Weaknesses

- A clear mentoring plan – i.e. specifics by which Dr. Sands and Dr. Moore will provide mentoring -- is not explicit
- Applicant is changing institutions just prior to this award. A clear discussion of how this transition will be managed would help assuage concerns that this change will not disrupt his productivity.

3. Research Plan:

Strengths

- Innovative area of investigation with potential for great impact. The idea of a "patient-centric" approach to quality and the introduction of a medical informatics platform are attractive.
- Interesting mix of qualitative research (i.e. focus groups) with a RCT with defined endpoints

Weaknesses

- Specific aim 1 - The specific aims are quite detailed, with several specific questions. However, the methods are not clear about the details about how these questions will be addressed. From where will the focus group patients be recruited? Based on the Human Subjects section, it appears that Mt. Sinai physicians will be interviewed through "semi-structured questionnaires"? As a result, it appears that the patients and physicians will not be a representative sample of patients and GI physicians across the country.
- Specific Aim 2 –The specifics about the quality of care metrics is not well described. Will these be adapted from the quality of care indicators in the appendix or a different set developed by the candidate through the focus groups? The proposal to integrate the candidate's web-based application into the EHR seems critical to the success of including provider data. This is a not insignificant undertaking and it is not clear that the overall institutional EHR has approved such integration and how it would interface with the patient's ability to enter their own data.
- Specific Aim 3 – the pilot data support the ability of patients to provide baseline information, but it is less clear whether there will be adequate adherence with the monthly updates.
- Is the trial intended for patients of any disease severity? It would seem that those with very mild or quiescent disease would have very little change in their data at the monthly update. This could affect compliance with monthly updates or at the very least reduce the likelihood of seeing any meaningful influence of the intervention over an 18 month period.
- Some overall discussion of the limitations of this approach to patient populations of lower technology literacy (or who lack computer access) would appear warranted. Will there be language-specific platforms (especially Spanish given the candidate's discussion that there will be a high representation of patients from "Spanish Harlem.)" How does that in turn impact how this type of approach could be used for broader national efforts at measuring quality if significant segments of the population cannot feasibly be captured?

4. Mentor(s), Co-Mentor(s), Consultant(s), Collaborator(s):

Strengths

- Complementary expertise between the primary mentor (Dr. Sands) and co-mentor, (Dr. Moore). Both are leaders in their fields and are clearly invested in the candidate.

Weaknesses

- Track record of mentors (i.e. prior mentees) would be helpful.
- Dr. Moore will be off-site, although given the candidate's strong relationship in working with her, this is a minor concern
- Although the candidate seems quite adept at informatics, he would likely still benefit from mentors with informatics expertise particularly in the integration of this web-based application with HER.

5. Environment and Institutional Commitment to the Candidate:

Strengths

- Extremely strong environment for IBD clinical work should provide good access top patients.
- Mt. Sinai seems to provide strong institutional commitment

Weaknesses

- Since he will be new to Mt. Sinai, it is difficult to know how personally invested the institution will be in his success

Protections for Human Subjects:

Acceptable Risks and Adequate Protections

Data and Safety Monitoring Plan (Applicable for Clinical Trials Only):

Acceptable

Inclusion of Women, Minorities and Children:

G1A - Both Genders, Acceptable

M1A - Minority and Non-minority, Acceptable

C3A - No Children Included, Acceptable

Vertebrate Animals:

Acceptable

Biohazards:

Not Applicable (No Biohazards)

Training in the Responsible Conduct of Research:

Acceptable

Select Agents:

Not Applicable (No Select Agents)

Resource Sharing Plans:

Not Applicable (No Relevant Resources)

Budget and Period of Support:

Recommend as Requested

THE FOLLOWING RESUME SECTIONS WERE PREPARED BY THE SCIENTIFIC REVIEW OFFICER TO SUMMARIZE THE OUTCOME OF DISCUSSIONS OF THE REVIEW COMMITTEE ON THE FOLLOWING ISSUES:

PROTECTION OF HUMAN SUBJECTS (Resume): ACCEPTABLE

INCLUSION OF WOMEN PLAN (Resume): ACCEPTABLE, 1A

INCLUSION OF MINORITIES PLAN (Resume): ACCEPTABLE, 1A

INCLUSION OF CHILDREN PLAN (Resume): ACCEPTABLE, 1A

SCIENTIFIC REVIEW ADMINISTRATOR'S NOTES: The proposed Training in the Responsible Conduct of Research was acceptable. The lack of a plan for Resource Sharing was unacceptable in critique 2. However, in critique 3 it was noted that a Resource Sharing plan was not applicable (no relevant resources).

COMMITTEE BUDGET RECOMMENDATIONS: The budget was recommended as requested. Recommended direct cost levels are estimated and are subject to further adjustment based on the Institute's standard budget calculation practices.

NIH has modified its policy regarding the receipt of resubmissions (amended applications). See Guide Notice NOT-OD-10-080 at http://grants.nih.gov/grants/guide/notice-files/NOT-OD-10-080.html.
The impact/priority score is calculated after discussion of an application by averaging the overall scores (1-9) given by all voting reviewers on the committee and multiplying by 10. The criterion scores are submitted prior to the meeting by the individual reviewers assigned to an application, and are not discussed specifically at the review meeting or calculated into the overall impact score. For details on the review process, see http://grants.nih.gov/grants/peer_review_process.htm#scoring.

MEETING ROSTER

Digestive Diseases and Nutrition C Subcommittee
National Institute of Diabetes and Digestive and Kidney Diseases Initial Review Group
NATIONAL INSTITUTE OF DIABETES AND DIGESTIVE AND KIDNEY DISEASES
DDK-C 1
June 27, 2012 - June 28, 2012

CHAIRPERSON

GHISHAN, FAYEZ KHALAF, MD
PROFESSOR AND HEAD
DEPARTMENT OF PEDIATRICS
UNIVERSITY OF ARIZONA
TUCSON, AZ 85724

MEMBERS

ABRAHAM, CLARA , MD *
ASSOCIATE PROFESSOR
DEPARTMENT OF INTERNAL MEDICINE
YALE UNIVERSITY SCHOOL OF MEDICINE
NEW HAVEN, CT 06520

ANTON, STEPHEN D, PHD *
ASSISTANT PROFESSOR
DEPARTMENT OF AGING AND GERIATRICS
UNIVERSITY OF FLORIDA, COLLEGE OF MEDICINE
GAINESVILLE, FL 32611

BERTHOUD, HANS-RUDOLF , PHD *
GEORGE H. BRAY PROFESSOR
NEUROBIOLOGY OF NUTRITION LABORATORY
PENNINGTON BIOMEDICAL RESEARCH CENTER
LOUISIANA STATE UNIVERSITY
BATON ROUGE, LA 70808

BLACK, JENNIFER D, PHD
PROFESSOR
EPPLEY INSTITUTE FOR RESEARCH IN CANCER
AND ALLIED DISEASES
UNIVERSITY OF NEBRASKA MEDICAL CENTER
OMAHA, NE 68198-595

BOONE, DAVID L, PHD *
ASSISTANT PROFESSOR
DEPARTMENT OF MEDICINE
UNIVERSITY OF CHICAGO
CHICAGO, IL 60637

BRAUN, JONATHAN , MD, PHD
PROFESSOR AND CHAIRMAN
DEPARTMENT OF PATHOLOGY
AND LABORATORY MEDICINE
UNIVERSITY OF CALIFORNIA, LOS ANGELES
LOS ANGELES, CA 90095

BRODY, JONATHAN , PHD *
ASSOCIATE PROFESSOR
DEPARMENT OF SURGERY
THOMAS JEFFERSON UNIVERSITY
JEFFERSON MEDICAL COLLEGE
PHILADELPHIA, PA 19107

CAMILLERI, MICHAEL L, MD
PROFESSOR
DIVISION OF GASTROENTEROLOGY AND HEPATOLOGY
DEPARTMENT OF INTERNAL MEDICINE
MAYO CLINIC
ROCHESTER, MN 55905

CARELS, ROBERT A, PHD *
ASSOCIATE PROFESSOR
DEPARTMENT OF PSYCHOLOGY
BOWLING GREEN STATE UNIVERSITY
BOWLING GREEN, OH 43403

CHAN, ANDREW T, MPH, MD
ASSOCIATE PROFESSOR
DEPARTMENT OF MEDICINE
MASSACHUSETTS GENERAL HOSPITAL
BOSTON, MA 02114

COHN, STEVEN M, MD, PHD *
PAUL JANSSEN ASSOCIATE PROFESSOR OF MEDICINE
SCHOOL OF MEDICINE
UNIVERSITY OF VIRGINIA
CHARLOTTESVILLE, VA 22908

COPPARI, ROBERTO , PHD *
DEPARTMENT OF INTERNAL MEDICINE
UNIVERSITY OF TEXAS SOUTHWESTERN MEDICAL
CENTER
DALLAS, TX 75390

DEUTZ, NICOLAAS E, MD, PHD *
PROFESSOR
DEPARTMENT OF HEALTH AND KINESIOLOGY
TEXAS A&M UNIVERSITY
COLLEGE STATION, TX 77843-425

FENDRICK, A MARK, MD *
PROFESSOR
DEPARTMENT OF HEALTH MANAGEMENT
AND POLICY
UNIVERSITY OF MICHIGAN
ANN ARBOR, MI 48109

HELMRATH, MICHAEL A, MD
PROFESSOR
DIVISION OF PEDIATRIC AND THORACIC SURGERY
CINCINNATI CHILDREN'S HOSPITAL MEDICAL CENTER
CINCINNATI, OH 45229

HORWITZ, BRUCE H, MD, PHD *
ASSOCIATE PROFESSOR OF PEDIATRICS
DEPARTMENT OF PATHOLOGY
BRIGHAM AND WOMEN'S HOSPITAL
BOSTON, MA 02115

KALARCHIAN, MELISSA A, PHD
ASSOCIATE PROFESSOR
WESTERN PSYCHIATRIC INSTITUTE AND CLINIC
PITTSBURGH, PA 15213

KARPEN, SAUL J, MD, PHD
ACTING PROFESSOR OF PEDIATRICS
DIVISION CHIEF, PEDIATRIC GASTROENTEROLOGY,
HEPATOLOGY, AND NUTRITION
EMORY UNIVERSITY
ATLANTA, GA 30322

KATZ, JONATHAN P, MD
ASSISTANT PROFESSOR OF MEDICINE
GASTROENTEROLOGY DIVISION
UNIVERSITY OF PENNSYLVANIA
PHILADELPHIA, PA 191046144

LADENHEIM, ELLEN E, PHD *
ASSOCIATE PROFESSOR
DEPARTMENT OF PSYCHIATRY AND BEHAVIORAL SCIENCES
JOHNS HOPKINS UNIVERSITY
BALTIMORE, MD 21205

LAYDEN, THOMAS J, MD *
PROFESSOR AND CHAIR
DEPARTMENT OF MEDICINE
UNIVERSITY OF ILLINOIS AT CHICAGO
CHICAGO, IL 606127323

LIANGPUNSAKUL, SUTHAT , MD *
ASSOCIATE PROFESSOR
DEPARTMENT OF MEDICINE
DIVISION OF GASTROENTEROLOGY/HEPATOLOGY
INDIANA UNIVERSITY
INDIANAPOLIS, IN 46202

MACK, CARA LYNN, MD
ASSOCIATE PROFESSOR
DEPARTMENT OF PEDIATRICS
CHILDREN'S HOSPITAL COLORADO
DENVER, CO 80218

MAHER, JACQUELYN J, MD
PROFESSOR
DEPARTMENT OF MEDICINE
UNIVERSITY OF CALIFORNIA, SAN FRANCISCO
SAN FRANCISCO, CA 94110

MARRERO, JORGE A, MD
ASSOCIATE PROFESSOR
DEPARTMENT OF INTERNAL MEDICINE
UNIVERSITY OF MICHIGAN ANN ARBOR
ANN ARBOR, MI 48109

NGUYEN, TOAN D, MD
PROFESSOR OF MEDICINE & CHIEF
DIVISION OF GASTROENTEROLOGY AND HEPATOLOGY
UNIVERSITY OF TENNESSEE HEALTH SCIENCES CENTER
MEMPHIS, TN 38163

RAUFMAN, JEAN-PIERRE , MD *
PROFESSOR AND HEAD
DIVISION OF GASTROENTEROLGY AND HEPATOLOGY
DEPARTMENT OF MEDICINE
UNIVERSITY OF MARYLAND
SCHOOL OF MEDICINE
BALTIMORE, MD 21201

SEVERSON, WILLIAM E, PHD *
DIRECTOR
SHARED RESOURCES
REGIONAL BIOCONTAINMENT LABORATORY
CENTER FOR PREDICTIVE MEDICINE
UNIVERSITY OF LOUISVILLE
LOUISVILLE, KY 40222

SMOOT, DUANE T, MD
PROFESSOR OF MEDICINE AND CHIEF
EDWARD S. HARKNESS CHAIR
DEPARTMENT OF INTERNAL MEDICINE
MEHARRY MEDICAL COLLEGE
NASHVILLE, TN 37208

SNETSELAAR, LINDA G, PHD
PROFESSOR AND CHAIR
DEPARTMENT OF EPIDEMILOGY
COLLEGE OF PUBLIC HEALTH
UNIVERSITY OF IOWA
IOWA CITY, IA 52242

STERLING, RICHARD KEITH, MD *
PROFESSOR OF MEDICINE
DIVISION OF GASTROENTEROLOGY, HEPATOLOGY AND NUTRITION
VIRGINIA COMMONWEALTH UNIVERSITY HEALTH SYSTEM
RICHMOND, VA 23298

STYNE, DENNIS MICHAEL, MD *
PROFESSOR AND CHIEF
PEDIATRIC ENDOCRINOLOGY
UNIVERSITY OF CALIFORNIA DAVIS CHILDREN'S HOSPITAL
SACRAMENTO, CA 95817

THYFAULT, JOHN P, PHD *
ASSOCIATE PROFESSOR
DEPARTMENT OF NUTRITION AND EXERCISE PHYSIOLOGY
UNIVERSITY OF MISSOURI SCHOOL OF MEDICINE
COLUMBIA, MO 65211

YULE, DAVID I, PHD *
PROFESSOR
DEPARTMENT OF PHARMACOLOGY AND PHYSIOLOGY
SCHOOL OF MEDICINE AND DENTISTRY
UNIVERSITY OF ROCHESTER
ROCHESTER, NY 14642

ZORILLA, ERIC P., PHD *
ASSOCIATE PROFESSOR
DEPARTMENT OF NEUROPHARMACOLOGY
THE SCRIPPS RESEARCH INSTITUTE
LA JOLLA, CA 92037

SCIENTIFIC REVIEW ADMINISTRATOR

WELLNER, ROBERT , PHD
SCIENTIFIC REVIEW OFFICER
REVIEW BRANCH, DEA, NIDDK
NATIONAL INSTITUTES OF HEALTH
BETHESDA, MD 208925452

GRANTS TECHNICAL ASSISTANT

GREEN, TOYIA I
EXTRAMURAL SUPPORT ASSISTANT
REVIEW BRANCH, NIDDK
NATIONAL INSTITUTES OF HEALTH
BETHESDA, MD 20892

PROGRAM REPRESENTATIVE

PODSKALNY, JUDITH M, PHD
PROGRAM DIRECTOR
TRAINING AND CAREER DEVELOPMENT PROGRAM
DIVISION OF DIGESTIVE DISEASES AND NUTRITION
NIDDK, NATIONAL INSTITUTES OF HEALTH
BETHESDA, MD 20892

* Temporary Member. For grant applications, temporary members may participate in the entire meeting or may review only selected applications as needed.

Consultants are required to absent themselves from the room during the review of any application if their presence would constitute or appear to constitute a conflict of interest.
