# Supplementary material for: Impact of the Mobile HealthPROMISE Platform on the Quality of Care and Quality of Life in Patients With Inflammatory Bowel Disease: Study Protocol of a Pragmatic Randomized Controlled Trial
Source: JMIR Res Protoc. 2015 Feb 18;4(1):e23. doi: 10.2196/resprot.4042 (PMC4376196; doi:10.2196/resprot.4042)
Supplement: Supplementary file 2 [file resprot_v4i1e23_app2.pdf]

Study ID #: HSM#13-00335 **Form Version Date:6/19/2013**

TITLE OF RESEARCH STUDY

Title: HealthPROMISE: A Cross-Institutional Patient-centric Model for Improving Quality of Care and Quality of Life

PRINCIPAL INVESTIGATOR (HEAD RESEARCHER) NAME AND CONTACT INFORMATION:

Name: Ashish Atreja, MD, MPH

Physical Address: 5 E, 98th street, 11th floor. FPA Practice

Mailing Address: 1 Gustave L Levy Place Box 1069, NY, NY 10029

Phone: 216-312-6655

WHAT IS A RESEARCH STUDY?

A research study is when scientists try to answer a question about something that we don't know enough about. Participating may not help you or others.

People volunteer to be in a research study. The decision about whether or not to take part is totally up to you. You can also agree to take part now and later change your mind. Whatever you decide is okay. It will not affect your ability to get medical care at Mount Sinai.

Someone will explain this research study to you. Feel free to ask all the questions you want before you decide. Any new information that develops during this research study which might make you change your mind about participating will be given to you promptly.

Basic information about this study will appear on the website http://www.ClinicalTrials.gov. There are a few reasons for this: the National Institutes of Health (NIH) encourages all researchers to post their research; some medical journals only accept articles if the research was posted on the website; and, for research studies the FDA calls "applicable clinical trials" a description of this clinical trial will be available on http://www.ClinicalTrials.gov, as required by U.S. Law. This Web site will not include information that can identify you. At most, the Web site will include a summary of the results. You can search this Web site at any time.

PURPOSE OF THIS RESEARCH STUDY:

The purpose of this study is to find whether a web-based patient self-assessment system (called HEALTHPROMISE) can allow you and your physicians to monitor your symptoms more closely and lead to better control of IBD.

You may qualify to take part in this research study because you have been diagnosed with Crohn's disease or Ulcerative Colitis (Inflammatory bowel disease, IBD) that is being managed by Mount Sinai Medical Center physicians.

Funds for conducting this research are provided by Crohn's and Colitis Foundation of America (CCFA) and an NIH grant.

LENGTH OF TIME AND NUMBER OF PEOPLE EXPECTED TO PARTICIPATE

Your participation in this research study is expected to last 24 months.

The number of people expected to take part in this research study at this site will be as many as 500 patients in a randomized clinical trial.

The total number of people expected to take part in this research study is the same since Mount Sinai Medical Center is the only site for this study.

DESCRIPTION OF WHAT'S INVOLVED:

If you agree to participate in this research study, the following information describes what may be involved.

- A physician or clinical-research coordinator will, based on a flip of a coin, decide whether or not to give you access to an application that may be used either on your Smartphone or computer. The group that does not receive the application will serve as the control. Instruction will be provided on how to use every feature of the application when putting information about your symptoms online.
- As a participant you will be expected to use the application from home, work, etc. Contact information will be provided if there are any features of the application that may be unclear to you.
- Prior to initiation of the study the physician or clinical-research coordinator will likely collect information about your medical history and background.
- Over the duration of the study approved Mount Sinai personnel may contact you to ascertain initial impressions on the application. Adjustments may be made over the course of the study in accordance with your feedback.
- Your doctor at Mount Sinai will have access to the information you input in the application and will be regularly alerted on your status if appropriately used. As such, your care team may contact you over the course of the study to discuss your symptoms or to advise for an appointment.
- There will be no laboratory testing, tissue collection, drug or device deployment as part of this study.
- As the study progresses any and all information will be de-identified before becoming part of a database, which will allow physicians at Mount Sinai to expand research in IBD and better understand whether applications can facilitate improved patient outcomes through increased awareness of one's health and better patient-physician communication.
- When the study first begins, half of the patients will receive the application and the other half will receive a control application for the purposes of evaluating effectiveness. However, at the conclusion of the first year, all patients will be offered the application.

  Study Timeline:

  1) Baseline visit:

     a. The research coordination will review the study with you and obtain an informed consent.

   b. The research coordinator will collect relevant background information and medical history as deemed necessary.

   c. Access to the application will be given to the appropriate group. Personal instruction and/or an educational video will be provided to ensure a familiarity with how to use the application when home or at a site other than the hospital.

2) Subsequent visits with your Physician.

   a. These visits should proceed as they normally would. Your physician may use the information you have added to the application to have discussions about your health and how to best proceed with treatment.

3) Additional Contact by members of the research team.

   a. This may be necessary as we try to incorporate your feedback on improving the usability of the application so that is easier for you and your physician to communicate between one another.

YOUR RESPONSIBILITIES IF YOU TAKE PART IN THIS RESEARCH:

If you decide to take part in this research study you will be responsible for the following things:

1) Learning to use the application appropriately.
2) Adding information about your recent symptoms on a regularly so that your doctor may keep track of how you are doing.
3) Scheduling appointments with your doctors and using the application when possible to communicate directly with your doctor.

COSTS OR PAYMENTS THAT MAY RESULT FROM PARTICIPATION:

Taking part in this research study may not lead to added costs to you except travel to the site. Additionally, we will be providing a $50 at the end of study after the patient completes the exit survey.

POSSIBLE BENEFITS:

It is important to know that you may not get any benefit from taking part in this research. Others may not benefit either. However, possible benefits may be generation of knowledge that will hopefully lead to a better understanding of using web-based platforms for guided self-management, enhancing patient-physician communication and improving patient outcomes.

REASONABLY FORESEEABLE RISKS AND DISCOMFORTS:

There are no physical risks associated with this study since there will not be any blood draw, procedure or new drug administrated as part of this study. Occasionally, some of the questions we will ask you as part of this study may make you feel uncomfortable. You may refuse to answer any of the

questions and you may take a break at any time during the study. There always exists the potential for loss of private information; however, there are procedures in place to minimize this risk. The study data will be hosted in a secured platform and access to data restricted.

OTHER POSSIBLE OPTIONS TO CONSIDER:

You may decide not to take part in this research study without any penalty. The choice is totally up to you.

IN CASE OF INJURY DURING THIS RESEARCH STUDY:

If you believe that you have suffered an injury related to this research as a participant in this study, you should contact the Principal Investigator, Dr. Ashish Atreja.

ENDING PARTICIPATION IN THE RESEARCH STUDY:

You may stop taking part in this research study at any time without any penalty. This will not affect your ability to receive medical care at Mount Sinai or to receive any benefits to which you are otherwise entitled.

If you decide to stop being in the research study, please contact the Principal Investigator or Research Coordinator. If you stop being in the research study, already collected information may not be removed from the research study database and will continue to be used to complete the research analysis. You may be asked whether the investigator can collect information from your routine medical care. If you agree, this data will be handled the same as research data.

You may also withdraw your permission for the use and disclosure of any of your protected information for research, but <u>you must do so in writing</u> to the Principal Investigator at the address on the first page. Even if you withdraw your permission, the Principal Investigator for the research study may still use the information that was already collected if that information is necessary to complete the research study. Your health information may still be used or shared after you withdraw your authorization if you should have an adverse event (a bad effect) from participating in the research study.

<u>Withdrawal without your consent</u>: The study doctor, the sponsor or the institution may stop your involvement in this research study at any time without your consent. This may be because the research study is being stopped, the instructions of the study team have not been followed, the investigator believes it is in your best interest, or for any other reason. If specimens or data have been stored as part of the research study, they too can be destroyed without your consent.

CONTACT PERSON(S):

If you have any questions, concerns, or complaints at any time about this research, or you think the research has hurt you, please contact the office of the research team and/or the Principal Investigator at phone number 216 312 6655

This research has been reviewed and approved by an Institutional Review Board. You may reach a representative of the Program for the Protection of Human Subjects at Icahn School of Medicine at Mount Sinai at telephone number (212) 824-8200 during standard work hours for any of the following reasons:

- Your questions, concerns, or complaints are not being answered by the research team.
- You cannot reach the research team.
- You are not comfortable talking to the research team.
- You have questions about your rights as a research subject.
- You want to get information or provide input about this research.

DISCLOSURE OF FINANCIAL INTERESTS:

None.

MAINTAINING CONFIDENTIALITY – HIPAA AUTHORIZATION

As you take part in this research project it will be necessary for the research team and others to use and share some of your private protected health information. Consistent with the federal Health Insurance Portability and Accountability Act (HIPAA), we are asking your permission to receive, use, and share that information.

<u>What protected health information is collected and used in this study, and might also be disclosed (shared) with others?</u>

As part of this research project, the researchers will collect your name, address, phone number, and email.
During the study the researchers will gather information by:

– We will directly measure whether your overall quality of life is improving over the course of study as a result of increased communication between you and your doctor. We will also separately measure your utilization of the application as well as physician engagement in making adjustments in response to updates on your status.

<u>Why is your protected health information being used?</u>

Your personal contact information is important to be able to contact you during the study. Your health information and the results of any tests and procedures being collected as part of this research study will be used for the purpose of this study as explained earlier in this consent form. The results of this study could be published or presented at scientific meetings, lectures, or other events, but would not include any information that would let others know who you are, unless you give separate permission to do so.

The research team and other authorized members of The Mount Sinai Hospital and Icahn School of Medicine at Mount Sinai (together, "Mount Sinai") workforce may use and share your information to

ensure that the research meets legal, institutional or accreditation requirements. For example, the School's Program for the Protection of Human Subjects is responsible for overseeing research on human subjects, and may need to see your information. If you receive any payments for taking part in this study, the Mount Sinai Medical Center Finance Department may need your name, address, social security number, payment amount, and related information for tax reporting purposes. If the research team uncovers abuse, neglect, or reportable diseases, this information may be disclosed to appropriate authorities.

Who, outside Mount Sinai, might receive your protected health information?

As part of the study, the Principal Investigator, study team and others in the Mount Sinai workforce may disclose your protected health information, including the results of the research study tests and procedures, to the following people or organizations: (It is possible that there may be changes to the list during this research study; you may request an up-to-date list at any time by contacting the Principal Investigator.)

- The United States Department of Health and Human Services and the Office of Human Research Protection.
- CCFA
- NIH

In all disclosures outside of Mount Sinai, you will not be identified by name, social security number, address, telephone number, or any other direct personal identifier unless disclosure of the direct identifier is required by law. Some records and information disclosed may be identified with a unique code number. The Principal Investigator will ensure that the key to the code will be kept in a locked file, or will be securely stored electronically. The code will not be used to link the information back to you without your permission, unless the law requires it, or rarely if the Institutional Review Board allows it after determining that there would be minimal risk to your privacy. We may publish the results of this research. However, we will keep your name and other identifying information confidential.

For how long will Mount Sinai be able to use or disclose your protected health information?

Your authorization for use of your protected health information for this specific study does not expire.

Will you be able to access your records?

During your participation in this study, you will have access to your medical record and any study information that is part of that record. The investigator is not required to release to you research information that is not part of your medical record.

Do you need to give us permission to obtain, use or share your health information?

NO! If you decide not to let us obtain, use or share your health information you should not sign this form, and you will not be allowed to volunteer in the research study. If you do not sign, it will not affect your treatment, payment or enrollment in any health plans or affect your eligibility for benefits.

Can you change your mind?

You may withdraw your permission for the use and disclosure of any of your protected information for research, but you must do so in writing to the Principal Investigator at the address on the first page. Even if you withdraw your permission, the Principal Investigator for the research study may still use your protected information that was already collected if that information is necessary to complete the study. Your health information may still be used or shared after you withdraw your authorization if you should have an adverse event (a bad effect) from being in the study. If you withdraw your permission to use your protected health information for research that means you will also be withdrawn from the research study, but standard medical care and any other benefits to which you are entitled will not be affected. You can also tell us you want to withdraw from the research study at any time without canceling the Authorization to use your data.

If you have not already received it, you will also be given The Mount Sinai Hospital Notice of Privacy Practices that contains more information about how Mount Sinai uses and discloses your protected health information.

It is important for you to understand that once information is disclosed to others outside Mount Sinai, the information may be re-disclosed and will no longer be covered by the federal privacy protection regulations. However, even if your information will no longer be protected by federal regulations, where possible, Mount Sinai has entered into agreements with those who will receive your information to continue to protect your confidentiality.

Signature Block for Capable Adult

Your signature below documents your permission to take part in this research and to the use and disclosure of your protected health information. A signed and dated copy will be given to you.

DO NOT SIGN THIS FORM AFTER THIS DATE →

_______________________________________ ____________________
Signature of subject Date

_______________________________________ ____________________
Printed name of subject Time

<u>Person Explaining Study and Obtaining Consent</u>

_______________________________________ ____________________
Signature of person obtaining consent Date

_______________________________________ ____________________
Printed name of person obtaining consent Time

<u>If the individual cannot read, a witness is required to observe the consent process and document below:</u>

My signature below documents that the information in the consent document and any other written information was accurately explained to, and apparently understood by, the subject, and that consent was freely given by the subject.

_______________________________________ ____________________
Signature of witness to consent process *Date*

_______________________________________ ____________________
Printed name of person witnessing consent process *Time*
