# Supplementary material for: Impact of the Mobile HealthPROMISE Platform on the Quality of Care and Quality of Life in Patients With Inflammatory Bowel Disease: Study Protocol of a Pragmatic Randomized Controlled Trial
Source: JMIR Res Protoc. 2015 Feb 18;4(1):e23. doi: 10.2196/resprot.4042 (PMC4376196; doi:10.2196/resprot.4042)
Supplement: Supplementary file 3 [file resprot_v4i1e23_app3.pdf]

CONSORT-EHEALTH Checklist V1.6.2 Report

(based on CONSORT-EHEALTH V1.6), available at [http://tinyurl.com/consort-ehealth-v1-6].

4042

Date completed

11/15/2014 16:03:06

by

Ashish

A Pragmatic randomized controlled trial to evaluate the impact of mobile HealthPROMISE Platform on the quality of care and quality of life in patients with inflammatory Bowel Disease

TITLE

1a-i) Identify the mode of delivery in the title

"mobile HealthPROMISE Platform"

1a-ii) Non-web-based components or important co-interventions in title

A "Pragmatic" randomized controlled trial. Effort will be optimized to enhance engagement of patients and providers.

1a-iii) Primary condition or target group in the title

" in patients with inflammatory Bowel Disease"

ABSTRACT

1b-i) Key features/functionalities/components of the intervention and comparator in the METHODS section of the ABSTRACT

" determine the impact of HealthPROMISE app in improving outcomes (quality of care, QOL, patient adherence, disease control and resource utilization) as compared to patient education app"

1b-ii) Level of human involvement in the METHODS section of the ABSTRACT

Pragmatic" randomized controlled trial.

1b-iii) Open vs. closed, web-based (self-assessment) vs. face-to-face assessments in the METHODS section of the ABSTRACT

"Participants will be recruited during face-face visits"

1b-iv) RESULTS section in abstract must contain use data

"The primary endpoint is number of quality indicators met in HealthPROMISE vs. control arm. Secondary endpoints include decrease in number of emergency visits due to IBD, Decrease in number of hospitalization due to IBD , change in generic QOL score from baseline, proportion of patients in each group who meet all eligible outpatient quality metrics and proportion of patients in disease control in each group. In addition, we plan to conduct protocol analysis of intervention patients with adequate HealthPROMISE utilization (> 6 logins from week 0- week 52) achieving above mentioned primary and secondary endpoints."

1b-v) CONCLUSIONS/DISCUSSION in abstract for negative trials

"we hope to show that. IBD patients who participate in their own care and share in decision-making have appreciably improved outcomes when compared to patients who do not."

INTRODUCTION

2a-i) Problem and the type of system/solution

Goal is to complement existing practices and EHR technology. Outlined in the introduction.

2a-ii) Scientific background, rationale: What is known about the (type of) system

Reasons for study and prior background is detailed in introduction.

METHODS

3a) CONSORT: Description of trial design (such as parallel, factorial) including allocation ratio

"Our hypothesis is that a patient-centric self-monitoring and collaborative decision support platform will lead to sustainable improvement in overall quality of IBD patients."

3b) CONSORT: Important changes to methods after trial commencement (such as eligibility criteria), with reasons

not applicable since this protocol paper

3b-i) Bug fixes, Downtimes, Content Changes

not applicable since this protocol paper

4a) CONSORT: Eligibility criteria for participants

Detailed in abstract and paper.Inclusion Criteria:

•Males and females aged above 18 years, inclusive

•Internet Access at home or Smartphone availability

•Ability to complete a web-based questionnaire in English language.

Exclusion Criteria

•Unable to communicate with the investigators and comply with the study requirements

•Presence of short bowel syndrome or stoma

•Presence of a condition or disease that, in the opinion of the investigators, may make it exceedingly difficult for the patient to use HealthPROMISE app, including, but not limited to, advanced dementia.

4a-i) Computer / Internet literacy

"Ability to complete a web-based questionnaire in English language"

4a-ii) Open vs. closed, web-based vs. face-to-face assessments:

"Patients will be enrolled through outpatient and inpatient facilities in an academic center"

4a-iii) Information giving during recruitment

through information paper and electronic flyers.

4b) CONSORT: Settings and locations where the data were collected

Online.

4b-i) Report if outcomes were (self-)assessed through online questionnaires

"All outcome data will be collected online. "

4b-ii) Report how institutional affiliations are displayed

recruited from patient pool

5) CONSORT: Describe the interventions for each group with sufficient details to allow replication, including how and when they were actually administered

5-i) Mention names, credential, affiliations of the developers, sponsors, and owners

authors developed the application. Mentioned in COI

5-ii) Describe the history/development process

Described in introduction and references to mockups and prior pilot included

5-iii) Revisions and updating

Development is frozen

5-iv) Quality assurance methods

This is a protocol submission

5-v) Ensure replicability by publishing the source code, and/or providing screenshots/screen-capture video, and/or providing flowcharts of the algorithms used

This is a protocol submission

5-vi) Digital preservation

This is a protocol submission

5-vii) Access

"Intervention and control apps will be provide free of charge to patients and patients will be given $25 after filling initial and end of study questionnaires. "

5-viii) Mode of delivery, features/functionalities/components of the intervention and comparator, and the theoretical framework

Wagners chronic disease framework mentioned.

5-ix) Describe use parameters

intervention patients have to use it every 2 weeks.

5-x) Clarify the level of human involvement

pragmatic nature allows us to increase level of effort if needed to meet process variables. We plan to report effort in final publication.

5-xi) Report any prompts/reminders used

patients and providers are alerted. pragmatic nature allows us to increase level of effort if needed

5-xii) Describe any co-interventions (incl. training/support)

Walkthrough of app will be done for patients as well as providers. Access to training video will be provided on app and provider dashboard.

6a) CONSORT: Completely defined pre-specified primary and secondary outcome measures, including how and when they were assessed

Yes, The primary endpoint will be number of quality indicators met in HealthPROMISE vs. control arm. The secondary endpoints will include:

•Decrease in number of Emergency visits due to IBD
•Decrease in number of Hospitalization due to IBD
•Change in generic QOL score (EQ5D) from baseline
•Proportion of patients in each group who meet all eligible outpatient quality metrics
•Proportion of patients in disease control in each group
•Emergency visits in each group
•Hospitalizations in each group
•General QOL scores in each group
•Per protocol analysis of intervention patients with adequate HealthPROMISE utilization (> 6 logins from week 0- week 52) achieving above mentioned primary and secondary endpoints

6a-i) Online questionnaires: describe if they were validated for online use and apply CHERRIES items to describe how the questionnaires were designed/deployed

references included for PAM 13 and eheals

6a-ii) Describe whether and how "use" (including intensity of use/dosage) was defined/measured/monitored

Submission is for protocol. we will measure intensity of dose/engagement

6a-iii) Describe whether, how, and when qualitative feedback from participants was obtained

Qualitative feedback (focus groups and delphi) were done in earlier phase. this protocol is for RCT

6b) CONSORT: Any changes to trial outcomes after the trial commenced, with reasons

not applicable.

7a) CONSORT: How sample size was determined

7a-i) Describe whether and how expected attrition was taken into account when calculating the sample size

30% attrition taken into account

7b) CONSORT: When applicable, explanation of any interim analyses and stopping guidelines

yes, interim analysis and stopping guidelines included

8a) CONSORT: Method used to generate the random allocation sequence

computer algorithm used to automate random allocation sequence

8b) CONSORT: Type of randomisation; details of any restriction (such as blocking and block size)

No blocking included.

9) CONSORT: Mechanism used to implement the random allocation sequence (such as sequentially numbered containers), describing any steps taken to conceal the sequence until interventions were assigned

Computer assigns randomization at the completion of online questionnaire. no manual input needed, so less chance of bias.

10) CONSORT: Who generated the random allocation sequence, who enrolled participants, and who assigned participants to interventions

automated computer algorithm

11a) CONSORT: Blinding - If done, who was blinded after assignment to interventions (for example, participants, care providers, those assessing outcomes) and how

11a-i) Specify who was blinded, and who wasn't

those assessing outcomes are blinded. Participants not aware of exact intervention they get since both groups get apps.

11a-ii) Discuss e.g., whether participants knew which intervention was the "intervention of interest" and which one was the "comparator"

Participant unaware for most part the exact intervention. They can guess, but its not explicit

11b) CONSORT: If relevant, description of the similarity of interventions

Both arms have apps. one is education app and other is disease monitoring app

12a) CONSORT: Statistical methods used to compare groups for primary and secondary outcomes
details outlined for entire statistical analysis plan in methods section including Chi Square, ANOVA.
12a-i) Imputation techniques to deal with attrition / missing values
participants have to complete entry and exit questionnaire to be included in study. Primary outcomes do not require imputation.
12b) CONSORT: Methods for additional analyses, such as subgroup analyses and adjusted analyses
Yes, subgroup analysis and adjusted analysis through ANOVA are also listed in methods section

RESULTS

13a) CONSORT: For each group, the numbers of participants who were randomly assigned, received intended treatment, and were analysed for the primary outcome
not relevant for protocol. we will list them in final report
13b) CONSORT: For each group, losses and exclusions after randomisation, together with reasons
we wil report them as in consort diagram when trial completed
13b-i) Attrition diagram
We have detailed plan for keeping track of login files and including as covariate
14a) CONSORT: Dates defining the periods of recruitment and follow-up
Yes, total study for 104 weeks with 52 weeks interim analysis
14a-i) Indicate if critical "secular events" fell into the study period
Not relevant for protocol submission
14b) CONSORT: Why the trial ended or was stopped (early)
Not relevant for protocol submission
15) CONSORT: A table showing baseline demographic and clinical characteristics for each group
Not relevant for protocol submission
15-i) Report demographics associated with digital divide issues
Not relevant for protocol submission
16a) CONSORT: For each group, number of participants (denominator) included in each analysis and whether the analysis was by original assigned groups
16-i) Report multiple "denominators" and provide definitions
Not relevant for protocol submission.
16-ii) Primary analysis should be intent-to-treat
yes, primary analysis is intent to treat
17a) CONSORT: For each primary and secondary outcome, results for each group, and the estimated effect size and its precision (such as 95% confidence interval)
Not relevant for protocol submission
17a-i) Presentation of process outcomes such as metrics of use and intensity of use
Yes, we have pre-specified process outcomes and metrics of use
17b) CONSORT: For binary outcomes, presentation of both absolute and relative effect sizes is recommended
Not relevant for protocol submission
18) CONSORT: Results of any other analyses performed, including subgroup analyses and adjusted analyses, distinguishing pre-specified from exploratory
Not relevant for protocol submission
18-i) Subgroup analysis of comparing only users
We also have subgroup analysis per protocol of users only
19) CONSORT: All important harms or unintended effects in each group
Not relevant for protocol submission. we plan to report after completion of trial
19-i) Include privacy breaches, technical problems
Not relevant for protocol submission. we plan to report after completion of trial
19-ii) Include qualitative feedback from participants or observations from staff/researchers
Not relevant for protocol submission. we plan to report after completion of trial

DISCUSSION

20) CONSORT: Trial limitations, addressing sources of potential bias, imprecision, multiplicity of analyses
20-i) Typical limitations in ehealth trials
We plan to report after completion of trial
21) CONSORT: Generalisability (external validity, applicability) of the trial findings
21-i) Generalizability to other populations
its generalizable platform and we have suggested the reasons. We plan to report more details after completion of trial
21-ii) Discuss if there were elements in the RCT that would be different in a routine application setting
More relevant to report after completion of trial
22) CONSORT: Interpretation consistent with results, balancing benefits and harms, and considering other relevant evidence
22-i) Restate study questions and summarize the answers suggested by the data, starting with primary outcomes and process outcomes (use)
Not relevant for protocol submission. we plan to report after completion of trial
22-ii) Highlight unanswered new questions, suggest future research
We have detailed this in future research section

Other information

23) CONSORT: Registration number and name of trial registry
Registration number is applied and we hope to get it in next 4 weeks
24) CONSORT: Where the full trial protocol can be accessed, if available
Once clinical trials.gov is available, we plan to make full protocol available at http://www.healthpromise.org/protocol (password protocol).
25) CONSORT: Sources of funding and other support (such as supply of drugs), role of funders
It mentions NIH funding and gives details of funding in supplement
X26-i) Comment on ethics committee approval
Informed consent attached.

x26-ii) Outline informed consent procedures
we are submitted informed consent document
X26-iii) Safety and security procedures
All platform is HIPAA complaint. end user will be guided for security and privacy protection. no data is stored in devices to protect against loss of privacy due to theft.
X27-i) State the relation of the study team towards the system being evaluated
The development is in-house and that is conflict of interest.
